# Social Support Experiences of Spousally Bereaved Individuals in a South African Township Community: The *Botho/Ubuntu* Perspective

**DOI:** 10.3389/fpsyg.2021.604987

**Published:** 2021-09-24

**Authors:** Tsholofelo Angela Thomas

**Affiliations:** Department of Psychology, University of Pretoria, Pretoria, South Africa

**Keywords:** bereavement, *botho*, spousal bereavement, *ubuntu*, widowhood, death, social support, interdependence

## Abstract

Bereavement is a deeply personal experience that is also shaped by one’s socio-cultural context. This qualitative study explored the social support experiences and needs of spousally bereaved individuals in a South African township. The *botho/ubuntu* philosophical framework was used to interpret participants’ experiences in this regard. Six ethnically diverse, bereaved spouses aged 55–67years, residing in a predominantly Setswana-speaking township in the North West Province of South Africa, were interviewed. Thematic analysis was used to analyze the data. An indigenous knowledge consultant was interviewed to situate participant experiences pertaining to mourning rites and traditions within the indigenous socio-historical and contemporary cultural context. The following themes were identified: (i) Sources of social support during bereavement; (ii) Inadequate social support after spousal death; (iii) The need for grief counseling; and (iv) Social restrictions and systematic isolation during the traditional mourning period: “It is as if you smell.” Broadly, bereaved spouses drew on their support networks at various stages of their bereavement, which included family members, in-laws, friends, burial societies, their surrounding communities, and religious communities and figures. However, some experienced ostracization and stigmatization during the mourning period, which was invariably longer for the widows in this study, in line with conventions across Black South African cultures. Some participants reported withdrawal of support by their in-laws and harmful attitudes and assumptions rooted in patriarchal ideology by family members and in-laws. As pertaining to *botho/ubuntu*, the study also showed that communality or relationality entailed both positive and negative aspects, including support, co-operation, care, lack of support, stigmatization, and ostracization. Unlike conventional conceptualizations of *botho/ubuntu*, the study findings illustrate the human experience as comprising varying dimensions of relationality, ranging from harmony to disharmony. Findings regarding the negative aspects of communality are compatible with those relating to relational interdependence in African and East Asian settings. The findings also expand our understanding of the nature of disharmony alongside harmony in interdependent socio-cultural contexts.

## Introduction

Losing a spouse to death is a life-changing event, with varying implications for survivors. In addition to grief over losing a loved one, bereaved spouses’ sense of loss may be compounded by multiple stressors stemming from spousal death, such as loneliness, taking on new roles and responsibilities (e.g., being a single parent and a breadwinner), and adjusting to the reality of losing a primary support system in the deceased spouse (e.g., Lowe and McClement, 2010; Ennis and Majid, 2020). Social support is one of the key resources for surviving spouses when coming to terms with spousal death. Lack of social support could predispose bereaved spouses to complicated grief, which is characterized by debilitating, prolonged bereavement (Lobb et al., 2010; Jordan and Litz, 2014). This study focuses on the social support received by bereaved spouses after a spouse’s death and their social support needs. Further, their experiences are interpreted according to the *botho/ubuntu* theoretical perspective.

### The Psychosocial Benefits of Bereavement Rituals

According to the traditional African worldview, death is a transition into eternal spiritual existence as an ancestor or “the living dead” (Baloyi and Makobe-Rabothata, 2014). Acknowledgement and remembrance of the deceased evolve along with their transcendence into the spiritual world, as demonstrated by the rites performed at different stages after their death. Beyond their cultural significance and therapeutic effects on the bereaved (Msimanga-Ramatebele, 2008, unpublished; Makgahlela et al., 2021), the cultural rituals performed after a spouse’s death play an important role in widows’ appraisal of the psychosocial support and care that they have received from designated elders (Msimanga-Ramatebele, 2008, unpublished). The latter signifies the importance of psychosocial support to bereaved spouses, and the manner in which its provision is played out in, or interlinked with cultural rites following a spouse’s death. In their study, Kokou-Kpolou et al. (2017a) have also demonstrated the significance of ritual practices to bereaved Togolese immigrants in Europe, based on whether they could participate in their loved ones’ funeral rituals in their countries of origin. A low score on complicated grief was found to be associated with participation in bereavement rituals for a loved one, while non-participation was associated with a high score on complicated grief. These authors concluded that participation in bereavement rituals alleviate bereaved persons’ grief and sense of loss, and reinforce family and social ties within the bereaved family and social group.

Bereavement rituals in Black South African cultures before and after the burial include bereaved spouses ingesting specially prepared herbs, shaving their heads, wearing mourning clothes, and undergoing ritual cleansing after the traditional mourning period elapses (Makgahlela et al., 2021). Ancestor reverence rituals also take place before and after the burial, and continue in varying forms long thereafter (Baloyi and Makobe-Rabothata, 2014; Makgahlela et al., 2021). Makgahlela et al. (2021, p. 97) acknowledge that these rituals hold multiple psychosocial benefits for the bereaved; among these, is ensuring that grief does not become “disabling and protracted” for the bereaved. On account of the integral role played by these rituals in bereavement, Yawa (2010, unpublished) posits that, while intrapersonal processes of bereavement are common in Western and Black South African cultures, interpersonal bereavement processes are equally dominant in the latter cultures. Interpersonal bereavement processes entail externalized, public expressions of mourning (Manyedi et al., 2003), such as bereavement rituals. In the broader sub-Saharan African context, Kokou-Kpolou et al. (2017a, p. 1249) also emphasize the importance of “the social and collective nature of bereavement” for Africans, which plays out in communal bereavement rituals.

### Social Support vs. Social Isolation During Bereavement

Other than engagement in cultural bereavement rituals as a mutually beneficial display of adherence to the social contract between the bereaved and their encompassing communities (Manyedi et al., 2003), bereaved spouses also draw from a variety of external resources to actively cope with their loss. These include peers, family, friends, and religious figures, who provide, among others, psychosocial support to the bereaved (Sekgobela et al., 2019). Community members also extend support to the bereaved family by paying visits to and performing chores in the bereaved household, respectively (Manyedi et al., 2003; Yawa, 2010, unpublished).

The above-mentioned support measures collectively make up social support to the bereaved. Uchino (2004, p. 10) has defined social support as inclusive of “both the *structures* of an individual’s life (for example, group memberships or existence of familial ties) and the more explicit *functions* they may serve (for example, provision of useful advice or emotional support)” or “structural support” and “functional support,” respectively. Social integration, demonstrated by individuals’ embeddedness within, and integration into a particular social network, forms the basis of the notion of structural social support. Integration into social networks bestows meaningful identities and presupposes certain social roles by all social actors within a particular social network (Uchino, 2004). In the context of this study, structural social support is considered to broadly comprise the existence of social relationships (e.g., relationships with friends and family, and membership to religious and other communities; Cohen and Wills, 1985) and the accessibility of these social networks or members thereof at the time of bereavement. The establishment of meaningful social networks and their accompanying roles or those of their members (Stryker and Burke, 2000) presupposes their provision of functional support to members (Cohen and Wills, 1985). In this study, functional support is considered to comprise social companionship and emotional, instrumental, and informational support (Cohen and Wills, 1985; Rook, 1987).

The types and sources of social support provided to bereaved persons typically evolve along with the period of grief. Soon after bereavement, the bereaved family’s support network across contexts (Somhlaba and Wait, 2009; Sekgobela et al., 2019) typically provides emotional and instrumental support (Powers et al., 2014), which comprise comforting the bereaved; helping with funeral arrangements; making financial and other material contributions; and tending to guests and performing chores around bereaved households. Powers et al. (2014) note that the support provided may diminish over time, with those providing the support assuming that the bereaved have gradually adjusted to the idea of having lost a spouse. This assumption may equally hold for bereaved spouses in a South African context. However, bereaved South African spouses’ psychological wellbeing may be undermined by not only their appraisal of diminished emotional and instrumental support over time, but may also be compounded by the systematic social isolation that they experience in the months following spousal death, during the traditional mourning period (Manyedi et al., 2003; Mathonsi et al., 2016). In the South African context and many parts of Africa, bereaved spouses tend to be systematically deprived of the benefits of social ties during a mandatory, traditional mourning period (Manyedi et al., 2003; Idialu, 2012; Mathonsi et al., 2016; Nyangweso, 2017). Widows specifically, also tend to be subjected to poor treatment in their immediate family contexts and the broader society during and after their mourning periods. Examples of such treatment are widow disinheritance and eviction from their marital homes by their in-laws, which occurs in rural and urban areas alike (Young, 2006; Murugani et al., 2014; Ndlovu, 2015, unpublished; Owusu, 2018, unpublished). Without recourse, widows and their surviving children may end up in a dire socio-economic state after a spouse’s death and lose their sense of security (Murugani et al., 2014). Widows are also often blamed by their in-laws for their husbands’ deaths, whereas this assumption does not routinely hold for widowers (Manyedi et al., 2003; Ndlovu, 2015, unpublished). Broadly, bereaved spouses’ wellbeing and grief course may be negatively affected by the withdrawal of social support and hostility from their social networks.

As shown above, social network variables have the potential to shape bereaved spouses’ grief course and experiences, and broadly, psychological wellbeing after spousal loss. This necessitates a study on bereaved spouses’ experiences of social support following the death of a spouse. Further, identifying their social support needs during this period would help inform interventions to better support bereaved spouses, in consideration of their sociocultural context and the support resources that are available to them.

### Social Support From the *Botho/Ubuntu* Perspective

The *botho/ubuntu* philosophy imposes the moral imperative on bereaved spouses’ encompassing communities “to exhibit humanness” (Metz, 2011, p. 537) toward the bereaved, by showing compassion toward, and helping them. This aligns with the recognition of one’s inherent interdependence with other social actors and, ultimately, promoting the common good. This study adopted an indigenous theoretical framework – *botho/ubuntu* – as a lens through which to appraise the social support experiences and needs of bereaved spouses.

There is a dearth of scholarly theorizations on grief and bereavement, and on death in particular, in the African context. Baloyi and Makobe-Rabothata (2014) and Nwoye (2015) attribute this to the continuing domination of Western approaches in the study of psychology, coupled with the presumed universal applicability of these approaches and theories across contexts, cultures, and peoples. This severely limits scholarly understanding of the realities of people in non-Western contexts. This study seeks to address this oversight, by focusing on spousally bereaved individuals’ experiences of social support and related needs in a South African context. Using the *botho/ubuntu* theoretical framework, the study also seeks to situate these experiences within participants’ socio-cultural context. Given the historical and continuing marginalization of indigenous knowledge in presumably global scientific discourse, this study will contribute toward the body of literature on the application of African cultural concepts in empirical research. The *botho/ubuntu* philosophy, its principles, and application as the theoretical framework in this study, are discussed in the “Theoretical Framework” section of this paper.

### Research Questions

The following research questions guided this study:

1. What are bereaved spouses’ experiences of social support following the death of a spouse in a South African township context?
2. What are the social support needs of bereaved spouses in a South African township context?
3. How does *botho/ubuntu* account for the social support experiences of spousally bereaved individuals?

## Theoretical Framework

The Setswana variation of the southern African aphorism, *Motho ke motho ka batho* (“I am because we are”), emphasizes interrelationships not only between individuals, but also between themselves and various entities. The aphorism underlies the concept of *botho*, which Ramose (2015, p.69) translates into English as “humanness” or “humanity.” *Botho* essentially characterizes relational personhood, with relatedness being considered an integral aspect of being human (Letseka, 2013). From this perspective, for adherents of *botho*, construction of the self is necessarily in relation to the broader community and/or society. LenkaBula (2008) and Chilisa et al. (2017) contrast *botho*’s emphasis on relationality as the essence of being, with the Western individualistic and autonomous conception of being, as embodied in Descartes’ statement, “I think, therefore I am.”

The aphorism, *Motho ke motho ka batho*, is translatable into, and applied in many other southern African languages, such as Kalanga (Chilisa et al., 2017), Shona, TshiVenda, Xitsonga, Sesotho, isiXhosa, and isiZulu (Dolamo, 2013; Magosvongwe, 2016). The more widely known variation of the *botho* philosophy is its equivalent in South African Nguni languages (e.g., isiZulu, isiXhosa, and isiNdebele), namely, “*ubuntu*.” The apparent universality of the *botho/ubuntu* philosophy in parts of southern Africa and, in particular, across South Africa, demonstrates its significance as an existential maxim for Black southern Africans.

The notion of *botho/ubuntu* is compatible with that of interdependence in cultural psychology, which assumes inherent interconnections between individuals (Markus and Kitayama, 2010, p. 423), such that they use the “thoughts, feelings, and actions of others with whom [they are] in relationship” as a primary referent. Adams and Dzokoto (2003) expand on the concept of interdependence, by distinguishing between collectivist and relational interdependence. Collectivist interdependence refers to “an experience of self that is connected with others as more-or-less interchangeable members of a group” (Adams and Dzokoto, 2003, p. 348), demonstrated by a deindividuated self with a collective, rather than personal identity. In contrast, a relational interdependent self entails an individuated self that assumes inherent relationality with others, such that the self is essentially “connected to others in a network of personal relationships” (p. 347). Arguments have been proffered for a characterization of individuals in African societies as exhibiting a relational interdependence, as opposed to collectivist interdependence (see Adams and Dzokoto, 2003).

*Botho/Ubuntu* is considered to primarily entail carrying oneself in a humane manner (i.e., “respectful and polite”; Ramose, 2015, p. 70) in relation to others, with both the individual and community cognizant of their interdependence and complementarity. In practical application, *botho/ubuntu* from this perspective involves acknowledgement of the rights of individuals and their encompassing collectives, and necessitates a sense of responsibility from these parties toward each other, and by extension, their environments (LenkaBula, 2008). In line with the sense of relatedness/relationality evoked by *botho/ubuntu*, in addition to mutual respect, the principle also entails mutual support, care, and compassion.

In the context of bereavement, in South Africa, displays of *botho/ubuntu*, as conventionally conceptualized, entail the support provided to those who are bereaved, including by immediate and extended family members, and by individuals and communities in one’s immediate social circles in various contexts (Somhlaba and Wait, 2009; Sekgobela et al., 2019). However, as discussed in the “Introduction” section, *botho/ubuntu* as described above is not indiscriminately extended to bereaved spouses within their socio-cultural contexts. They often experience social isolation and stigmatization during their mandatory traditional mourning periods (Manyedi et al., 2003; Idialu, 2012; Mathonsi et al., 2016; Nyangweso, 2017), while widows are often subjected to poor treatment by their in-laws and communities (Manyedi et al., 2003; Young, 2006; Dlukulu, 2010, unpublished; Murugani et al., 2014; Ndlovu, 2015, unpublished; Owusu, 2018, unpublished). Such stigmatization is clearly constructed within a communal and societal context, filtering down to conventionalize stigmatizing attitudes and mourning traditions, beliefs, and practices pertaining to bereaved spouses in general. *Botho/Ubuntu* as conventionally constructed does not account for these. When considering LenkaBula’s (2008) above-mentioned reference to *botho* as entailing acknowledgement of individuals’ and their encompassing collectives’ rights and mutual accountability and responsibility among these parties, then these practices clearly disregard bereaved spouses’ rights, while favoring the ideals of the collective. Mourning rites and traditions also necessitate a sense of responsibility from those bereaved, whereas this is not consistently required of bereaved persons’ encompassing communities, as shown by the culture of stigmatization and victimization of bereaved spouses. Addressing this contradiction within the conventional conceptualization of *botho/ubuntu* as described above, Mboti (2015) has proposed a conceptualization of *botho/ubuntu* that does not limit the quality of humanness to harmonious relationships. He argues that human relationships entail both harmony and disharmony. As such, a conceptualization of *botho/ubuntu* that elevates harmony as the pinnacle of humanness, and disregards disharmony, does not accurately reflect what it means to be human in an African context. Mboti (2015, p. 141) further challenges us “to imagine instances where broken relationships are as authentically *human* and *humanizing* as much as harmonious relationships.” While interdependence in African (Adams and Dzokoto, 2003) and East Asian (Liu, 2020, unpublished) cultures is conventionally associated with cooperative relationships and communal harmony, within-group competition, mistrust, and covert interpersonal hostility or assumptions thereof, have been found to be rife in these contexts. These observations are compatible with Mboti’s (2015) notion of humanness as entailing both harmony and disharmony.

In the current study, in line with Mboti’s (2015) proposed conceptualization of *botho/ubuntu*, in the interpretation of the study findings, equal importance is ascribed to harmonious (including co-operation, compassion, and respect in relation to the bereaved spouse) and disharmonious relationality and relatedness, as pertaining to bereaved spouses’ experiences of social support and related needs during bereavement. The ways in which either dimension plays out in the social support experiences of participating bereaved spouses during the course of their bereavement will also be considered.

## Materials and Methods

### Research Approach

Qualitative methodology, drawing from an interpretivist paradigm, was used in this study. Qualitative methodology, as used in this study, is concerned with how individuals experience phenomena, in an attempt to explain these (Willig, 2008). Interpretivism emphasizes an understanding of the social world based on social actors’ interpretation of their reality, which is invariably relative (Bryman, 2016). Bryman (2016) adds that subscription to the interpretivist paradigm entails double interpretation, with the researcher also interpreting research participants’ interpretation of their reality. While this study’s focus is on individuals’ subjective experiences of social support during spousal bereavement, the researcher acknowledges that these experiences and perhaps interpretation thereof occur in a communally and culturally constructed context. The current study’s *botho/ubuntu* theoretical framework, as conceptualized from Mboti’s (2015) perspective, denotes both a constructionist and subjectivist epistemological stance in its emphasis on both relationality and individuality. Constructionism is defined as “the construction of knowledge through active interaction with environments” (Graue and Karabon, 2013, p. 13), such that meaning is constructed through social interaction. A subjectivist stance emphasizes the understanding of one’s reality due to individual, personal experience. The assumption is that in the current study context, though personal, bereaved spouses’ experiences and interpretation thereof are shaped by their cultural contexts and related conventions, to some degree.

In line with the notion that the researcher’s role essentially entails interpreting participants’ own interpretations (Bryman, 2016), I acknowledge that my positionality as the researcher, as shaped by my cultural and social background, and personal experiences, informed not only my interpretation of participants’ experiences and understanding of their interpretations, but also my general approach toward this study. I am a Black South African woman, socialized primarily in a Setswana and, to some extent, isiXhosa linguistic context, and secondarily in multi-lingual, multi-cultural, and multi-ethnic contexts in South Africa. I grew up in the township in which the bereaved participants reside. My role during the research process oscillated between that of an insider and outsider. I am familiar with the study site’s cultural context and the cultural practices associated with spousal bereavement, and I know some of the bereaved participants well as my elders. However, I was discernibly an outsider, insofar as my lack of first-hand experience of spousal death, coupled with the bereaved participants’ status as elders in relation to me in our cultural context.

### Participants

Snowball sampling was used as a sampling technique. This involved approaching several individuals from the target population, who expressed interest in participating in the study, then asking them to refer me to other individuals who met the study’s inclusion criteria (Laher and Botha, 2012). A sample of six participants who met the inclusion criteria and agreed to participate in the study was identified. The inclusion criteria were as follows: (i) Race: Black; (ii) Nationality: South African; (iii) Having experienced the death of a spouse within no less than 1year prior to this study; and (iv) Aged 21years or older.

Participating spousally bereaved individuals were five women and one man, who had lost their spouses to death for periods ranging from 2 to 12years at the time of the study. Participants’ ages ranged from 55 to 67years. Participants’ socio-demographic and bereavement characteristics are presented in detail in Table 1. The participants are residents of Ikageng township in the North West province of South Africa. The township and the North West province as a whole are predominantly Setswana-speaking areas. Despite this, the township is ethnically diverse, which essentially renders it a multi-lingual community within the context of South Africa.

**Table 1:** Participants’ socio-demographic and bereavement characteristics.

Pseudonym	Age in years	Gender	Time since loss	Cause of spouse’s death
MaLerumo	65	Female	2years	Chronic illness
MaMqwathi	61	Female	12years	Homicide
Masechaba	63	Female	9years	Recurring illness
MaTholo	55	Female	5years	Chronic illness
Nowam	67	Female	3years	Chronic illness
Phala	67	Male	3years	Acute illness

The inclusion criteria for the indigenous knowledge consultant was that they be knowledgeable about, and have lived experiences of indigenous mourning-related rites and practices within the South African context.

The indigenous knowledge consultant is a Motswana and identifies as an indigenous knowledge activist. Similar to the spousally bereaved participants, the consultant is a long-term resident of the North West province and could therefore provide insight into cultural rites and practices relating to spousal bereavement in the participating bereaved spouses’ geographical and socio-cultural context.

### Materials

Semi-structured interviews were used to collect data from bereaved spouses and the indigenous knowledge consultant. Interview guides were used to guide the interviews. To enhance mutual meaning-making between participants and the researcher, a colleague who is fluent in English and Setswana, to whom the participants’ cultural context is familiar, verified the accuracy of the translation of the interview guides from English to Setswana. All interviews were conducted predominantly in Setswana. In their responses, participants used Sepedi, some Sesotho, and mainly Setswana or mixtures of these, with variations in dialect presumably because of their linguistic backgrounds and geolinguistic differences. All analyzed data were subsequently translated into English as efficiently as possible; the above-mentioned colleague also verified translation of key Setswana concepts into English at this stage. Nonetheless, the researcher acknowledges the limitations in wholly preserving the integrity of participants’ meanings, as the English language proved to be limited in capturing nuances of the languages in which the interviews had been conducted. Thus, where necessary in the excerpts provided in the Results section, terms or phrases that are not readily translatable into English are provided in the original language used in interviews, along with explanations in English.

### Procedures

Ethical clearance was obtained from the Faculty of Humanities’ Ethics Committee at the University of Pretoria (Ref no: HUM030/1119). Participants received a consent form, one copy in Setswana and another in English, and an information sheet, providing details of the study and the study procedures. Participants provided written informed consent.

In interviews conducted in March 2020, bereaved spouses were asked questions pertaining to their: (i) Experiences following a spouse’s death; (ii) Reflections on their grief processes following the spouse’s death; (iii) Adherence to any culturally mandated conduct and practices following the spouse’s death; and (iv) Means of coping with the death of their spouses. The interview comprised questions such as: (i) What has helped you cope with the death of your spouse?; and (ii) What kind of support did you receive when you were grieving?

After data collection, the interview data were transcribed with the help of a research assistant who is a Setswana language practitioner. Thereafter, reflexive thematic analysis was used to analyze the data. Reflexive thematic analysis is a qualitative data analysis method involving the development of themes, based on patterns of shared meaning regarding a particular concept in a given data set (Braun and Clarke, 2020). This process entailed familiarization with the data through reading the transcripts and noting initial ideas regarding possible patterns in the data, followed by coding the data, generating initial themes from the codes, and developing and reviewing themes as needed (Braun et al., 2018; Braun and Clarke, 2020). Reflexive thematic analysis emphasizes the active role played by the researcher in reflectively and thoughtfully engaging with the data and generating themes from these, also acknowledging their subjectivity. Braun and Clarke (2019, p. 14) further posit that the generation of themes occurs “at the intersection of the researcher’s theoretical assumptions, their analytic resources and skill, and the data.” These assumptions are compatible with the current study’s subscription to the interpretivist paradigm. Use of the interpretivist paradigm in this study presupposes “double interpretation” (Bryman, 2016, p. 28), such that the findings obtained inevitably result from participants’ interpretations of their reality and my interpretation of those, which in turn, is essentially shaped by my own varying positionings (Braun et al., 2018), as indicated in the “Research Approach” section. Although my knowledge of participants’ cultural context enhanced my understanding of aspects of their experiences, particularly as relating to their cultural contexts, in some respects, it also limited my interpretation of their experiences, such that it could only occur within the confines of my cultural perspective.

I consulted some participants at various stages of the data analysis process to verify my understanding of their accounts, and to solicit elaboration on certain concepts. After analysis, I conducted member-checking by presenting reviewed themes to participants (Birt et al., 2016).

The semi-structured interview with the indigenous knowledge consultant took place in August 2020. In line with his expertise, the consultant was asked questions pertaining to his knowledge of contemporary and historical customs, rites, and rituals pertaining to the death of a spouse among the Batswana of South Africa. The data from this interview was subsequently transcribed. The indigenous knowledge consultant’s data were used to situate relevant themes or accounts – as identified from the data provided by bereaved spouses – within the contemporary and historical indigenous cultural context. In this paper, such situation was done particularly for themes relating to mourning rites and practices that bereaved spouses reported engaging in.

## Results

I identified four themes pertaining to spousally bereaved participants’ experiences of social support and social support needs, following their spouses’ deaths. To ensure anonymity, participants are referred to by pseudonyms. Explanatory terms in the excerpts supporting the themes below are in square brackets.

### Theme 1: Sources of Social Support During Bereavement

Sources of support for spousally bereaved participants ranged from the immediate and extended family, neighbors and acquaintances, to burial societies, employers, and to a large extent, church.

Among those who were employed at the time of their spouses’ death, some received moral and material support from their employers.

At least four participants reported church and fellow congregants as an important source of support. Traditionally, fellow congregants and church elders hold prayer sessions that are also attended by members of the community and acquaintances in the days preceding the funeral. The study participants described this and the support that they received through church long after the funeral, as having helped them come to terms with their grief.

Mme MaMqwathi described the significance of church in helping her through her grief as follows:

“*If you do not attend church, you are just sitting, isn’t it? You become overwhelmed by these things, you think about them … but now, when you are in church, you shed the heaviness, you become lighter*.” From this perspective, finding solace in church subscribes to Stroebe et al.’s (2005) reference to distraction from the loss being an important coping mechanism.

Mme MaMqwathi proceeded to explain the key role that her parish catechist played in helping her come to terms with her grief: “*Ntate Mogale* [parish catechist] *… ooh … he really built me. Ntate Mogale sat me down, he built me, it’s as if he was counseling me […] he is the one person who gave me strength*.”

Nowam reported receiving support from burial societies soon after her husband’s death. Burial societies are found all over Black South African communities. Their primary purpose is to systematically enable provision of moral, financial, and material support to their bereaved members. Members of burial societies make either monthly financial contributions or smaller financial contributions when a member’s dependant has died; and cater to people who come to pay their respects in the days before, and on the day of the funeral. Once someone’s death is announced, guests come to pay their respects and comfort the bereaved family on a daily basis until the day of the funeral. The largest number of guests received is on the day of the funeral. Hosting these guests throughout a given week or two requires many resources, as well as people to help perform the required chores around the bereaved household (e.g., cleaning and serving the guests). These duties are performed by family members, close acquaintances, and burial society members.

Nowam explained the impact of the support that she received from burial society members and acquaintances as follows:

“*They supported me, I even felt that I have people. Even when my family was far, I was not alone. They kept coming, one-by-one, but these people … they stood by me. Even now, I’m still … I saw how much they love me*.”

Neighbors, friends, and acquaintances supported bereaved families through measures such as helping around the household in the period preceding the funeral. One participant reported that a family friend assisted her with transportation to and from church and medical appointments during the mandatory mourning period that restricted her mobility beyond her home for months after the burial.

In the context of familial relationships, participants received support from their extended families, and others, from their in-laws. The support from both immediate and extended family, coupled with their constant presence in the days preceding the burial, helped participants feel less alone and “lost.” In the early period of bereavement, some strained relationships with in-laws gave way to compromise and co-operation between the bereaved spouse and in-laws, and the provision of material and moral support to the spouse.

Participants continued to receive support in some form or another from family after the funeral service. Nowam, who was 63years old at the time of her husband’s death, received prolonged support from extended family thereafter; a younger, adult family member (Keneilwe) resided with her for 6months, keeping her company. Keneilwe also solicited support on Nowam’s behalf when necessary:

“*It turns out that Keneilwe had been talking to [my friend] […], she told me after a long time that [Keneilwe] would tell her, ‘It seems that she misses Ntatemogolo; today she is crying. Please do not abandon her, Mme MaSerero, visit her sometimes’*.”

Within their immediate family environments, in the period following the spouse’s burial, a number of participants received support from their children, and some from their siblings. Children who resided with the bereaved spouses were a consistent source of emotional support for the bereaved during this period. It would therefore seem that immediate family members and those residing with the bereaved spouse in the household were best positioned to provide or facilitate the provision of structural social support and emotional support for a prolonged period after the spouse’s burial.

### Theme 2: Inadequate Social Support After Spousal Death

The study participants reported on the inadequacy of the support that they received from their social networks and their intensified grief when they had no access to social support at various stages of their bereavement. They also recounted instances that illuminated their spouses’ deaths and, along with these, the loss of certain forms of social support that are inherent to the marital social structure. These are discussed in the sub-themes below.

#### Inadequate Social Support From Existing Social Networks

Although it is common practice for especially close acquaintances to support bereaved persons and families in varying ways, some participants reported not receiving support from some parties, contrary to their expectations or wishes.

After receiving support from extended family and in the form of prayer sessions held prior to his wife’s funeral service, Ntate Phala’s only source of emotional support thereafter was his daughter. He recalled feeling “lost” during this period. Several other participants recounted feeling a pronounced sense of loss particularly in the days after the funeral, after everyone had left.

Limited access to support following the burial might have been compounded by the bereaved spouses’ help-seeking behaviors. For instance, Nowam concealed her grief from her children, fearing that this would trigger their own grief and deepen their concern for her. In turn, this limited her access to emotional support when needed.

Some participants reported not receiving support from their in-laws, contrary to expectations. In some instances, bereaved spouses received hostile treatment from in-laws. For instance, Mme MaTholo’s in-laws withheld their emotional and financial support, reasoning that because she alone had benefited financially from her husband while he was alive, she would have to figure out how to proceed without their assistance. As previously explained, because of the vast numbers of people who come to pay their respects, Black South African funerals tend to be costly. Therefore, it is customary for individuals or communities with which the bereaved family is affiliated, to contribute toward those costs; one of these ways is by making financial contributions toward the bereaved family or *go thusa ka matsogo* (i.e., “to lend a hand” however one can).

Illustrating hostile treatment toward the bereaved spouse, Mme MaMqwathi’s in-laws withheld their support and blamed her for her husband’s death: “*My in-laws were quite distant. You know, though, that when a husband has died, the wife always has a part [in that]; they think that you have a part, you played a role in the husband’s murder*.”

Contrary to the experiences of other participants in relation to support by their employers at the time, Mme Masechaba’s employer pressured her to return to work 6days after her husband’s death. She was distressed as a result, perceiving this to demonstrate her employer’s lack of empathy and disregard for her circumstances.

#### Implications of the Loss of Spousal Social Support After Spousal Death

As the bereaved spouses settled into life without their spouses, the vacant roles that the deceased spouses had left became illuminated. Spousal death deprived them of the benefits and forms of support mutually provided within the marital social structure (Uchino, 2004). Some of the issues that they faced due to the loss of spousal social support were intimacy needs, having no one to assist with the running of the household, lack of emotional and practical support when encountering life difficulties, and financial struggles.

Mme MaLerumo reported that surviving spouses did not have anyone to call on for help when struggling financially:

“*Okay, sometimes then, when you do not work, you will maybe say, ‘I am struggling […] What am I going to do?’ […] Then maybe you should … I don’t know … where to go, I don’t know where you can go. Right then, I don’t even know where you are supposed to ask for help … who are you going to ask for help?*”

Nowam reported missing her husband nearly 3years after his death, “*especially when I am struggling*.” Another participant, Mme Masechaba, reported feeling that her sense of survival was threatened, due to her household finances being under tremendous strain after her husband’s death. Aged 63years at the time of the study, Mme Masechaba was hopeful that her financial struggles would be eased once she reaches the age of 65, at which she would be eligible for an old-age government pension grant.

Illustrating the loneliness that spousal death predisposes surviving spouses to, the youngest of the study participants stated that one of the now unattainable benefits of an intimate relationship was, “*Just to chat with someone your age*.” In relation to this, the participant acknowledged struggling with unmet intimacy needs after her husband’s death.

### Theme 3: The Need for Grief Counseling

Based on their experiences, participants advocated for grief counseling for bereaved persons. One participant who was already providing support to widowed spouses advocated specifically for peer counseling:

“*Now really, I help a lot of people who are going through what I have gone through. […] If they [bereaved spouses] received counseling from other people who have lost their spouses. You see? So that they can show them that, ‘I have also been here*’” (Ntate Phala).

Mme MaTholo noted that, beyond the needs of the bereaved spouse, children could also benefit from counseling. She noted that children’s loss often went unacknowledged, with the surviving parent being the primary recipient of social support and consideration by others:

“*[…] when people come, they say, ‘Oh, MaTholo, I am so sorry!’ So, no one talks to those children. […] They have also experienced loss […]*.”

Mme MaTholo had also reported that her relationship with her children had deteriorated since her husband’s death. Her reference to the need for grief counseling for children after parental death might have resulted from her deduction that her children had not received the necessary support to cope with their father’s death. From this perspective, the resolution of their grief could also improve overall family dynamics and strengthen mutual structural social support for all members of the household. Mme MaTholo’s suggestion of grief counseling for bereaved children was significant, considering that other participants acknowledged the high likelihood of a change in family dynamics after a spouse’s death, with children likely to challenge the remaining spouse’s parental authority.

### Theme 4: Social Restrictions and Systematic Isolation During the Traditional Mourning Period: “It Is as if you Smell”

During the traditional mourning period, bereaved spouses were required to adhere to mandatory social restrictions. This period was marked by relative isolation for the bereaved and, at the extreme end, stigmatization and ostracization. After the traditional mourning period, the bereaved spouses underwent mandatory traditional cleansing, after which they could fully be reintegrated into their communities. These experiences are presented in the sub-themes below.

#### Mourning Clothes and Systematic Isolation During the Traditional Mourning Period

The traditional mourning period typically lasts from about 2–3months to a year. This period is marked by bereaved spouses’ relative confinement and being dressed in mourning attire. Participants’ mobility beyond the household was confined to commuting to and from work. The participant accounts illustrate the ways in which practices associated with the mourning period systematically drove bereaved spouses into virtual seclusion and predisposed them to stigmatization.

Traditionally, widows are required to wear *thapo* (i.e., “mourning attire/clothes”) or *bontsho* (i.e., “black”) to signify mourning. This has typically entailed being fully dressed in black attire, particularly for women. The indigenous knowledge consultant stated that wearing black to signify mourning is, in fact, a colonial phenomenon, since it suggests a period in which black dye could be acquired to produce black fabric and, therefore, black mourning clothes. In recent years, widows tend to wear *seshweshwe*, which is traditional Setswana/Sesotho attire, instead of black attire. The indigenous knowledge consultant attributed this to societal change and along with it, a change in what is considered of value by society at any given point. He extended this flexibility to the fact that, unlike widows, widowers tend to wear less distinctive clothing to signify mourning, and have shorter mourning periods. He attributed these concessions to a patriarchal social structure and the migrant labor system, wherein mostly Black men in South Africa’s apartheid-era Bantustans would work in urban areas previously demarcated as White South Africa, for more than 11months at a time (Ngwane, 2003; Cox et al., 2004). This, according to the indigenous knowledge consultant, rendered traditional practices relating to mourning impractical for these men, leading to concessions such as shorter mourning periods and less distinctive mourning attire.

During their mourning periods, some of the female study participants had worn black or dark-colored attire; others wore a black or dark shawl on their shoulders or head-covering only; and others had agreed with their husbands prior to their deaths that neither would wear black when the other died. This account of differences in attire signifying mourning is given so as to illustrate how bereaved spouses’ experiences of stigmatization, as described below, likely differed as a direct result of these obvious differences in attire, with those most noticeably dressed in mourning attire being most likely to attract negative public attention.

With regard to other people’s attitudes and behaviors during their mourning periods, participants gave accounts of varying degrees of stigmatization, and at its extreme end, social ostracization:

“*[…] even when I went to work, there are people who would treat you poorly; there are those who would treat you well. But … for the most part, I did not get those* [instances of poor treatment]” (Mme MaMqwathi).

Mme MaMqwathi was dressed in a black shawl over her shoulders during her mourning period, as opposed to full-on black attire. This might explain the relatively better treatment that she received in public, especially considering Nowam’s experiences, who wore dark-colored mourning attire from head to toe:

“*The thing is … some people … like … they say you are contaminating them with sefifi [the shadow of death]. You are bereaved […] you contaminate them with bad luck. So, even in taxis, when you are bereaved, you are not supposed to get into a taxi, especially not in front. In a taxi, someone else will move so you can sit, someone who is also a woman who is like you, who is widowed. […] as for when you are wearing black cloths, people do not want to sit next to you*.”Participants indicated that such treatment was hurtful.

Although not dressed in black like the spousally bereaved women, Ntate Phala also reported that, when wearing mourning clothes, “[…] *it is as if you smell. […] When people see you, not even a single one wants to hold your hand*.” Elaborating on their ostracization during the mourning period, he added that bereaved spouses inevitably become “*like something that has been discarded*.”

The above accounts align with the explanation given by the indigenous knowledge consultant, that mourning clothes indicate that one is accompanied by the shadow of death or darkness (*sefifi*) until the cleansing ceremony takes place. *Sefifi*, as presumably carried by a bereaved person, is assumed to have the potential to contaminate those around the bereaved with bad luck. This stance also seemingly justifies the requirement of minimal interaction with others outside the home by bereaved spouses.

Participants’ mobility restrictions were relaxed as the mourning period progressed. For instance, Nowam was able to attend church in the fourth month of her mourning period, after being confined to her home for 3months. Participants also unanimously noted that it was customary for women to be confined for longer periods.

#### Cleansing to Mark the End of the Traditional Mourning Period

Assumptions regarding *sefifi* and mourning clothes and the systematic isolation of bereaved spouses persist throughout the mourning period, until the bereaved person undergoes traditional cleansing. All participants underwent a traditional cleansing ceremony to mark the end of their mourning periods.

After the cleansing ceremony, bereaved persons are no longer required to wear mourning clothes. The only male participant underwent traditional cleansing after 3months of mourning. The female participants’ mourning periods were typically longer; the cleansing ceremonies of four of the five participating women took place 6months after the spouse’s death. The fifth woman was given concession for a shorter mourning period because she felt too physically weak to symbolically mourn for longer:

“*[…] I wore thapo [mourning clothes] for 4months. […] My problem was that I felt that … I was weak, I am not going to manage. Then, they said, ‘Because you are starting to get sick, this and that … So, we will not keep you for long’*” (Mme Masechaba).

The traditional cleansing ceremony marked the end of the bereavement period and participants’ full reintegration into their communities and society. After the ceremony, the bereaved spouses could resume unrestricted social relationships and interactions with members of their encompassing communities. Ntate Phala illustrated this reintegration as follows: “*After taking off the bereavement clothes, it is then that people will say, ‘Greetings,’ ‘Greetings,’ only then wanting to come closer to you*.”

## Discussion

Bereaved spouses in this study had varied experiences of social support. These ranged from the provision of social support by multiple sources, to deliberate withdrawal of support by members of their social networks and social isolation and stigmatization in the broader social context during their mourning periods. A change in household dynamics and the loss of spousal social support due to bereavement also had varying implications on the bereaved spouses and families. These are discussed in detail below. An appraisal of these varying dynamics from the *botho/ubuntu* perspective is also provided.

### Social Support Received During Bereavement

Bereaved spouses received structural and functional social support in various forms and from multiple sources. Shortly after the spouse’s death until the burial, the bereaved received a combination of structural, emotional, instrumental, and spiritual support from their neighbors, acquaintances, and surrounding communities; employers; religious communities; friends; and family, including their children, siblings, in-laws, and extended family members. Social support has been cited as a key resource for the bereaved after experiencing the death of a loved one, with the potential to avert prolonged psychological distress and dysfunctional bereavement reactions (Lobb et al., 2010; Jordan and Litz, 2014). In the South African context, Sekgobela et al. (2019) have highlighted the importance of psychosocial support to widows by various sources, including peers, family and friends, and religious figures. Similarly, Somhlaba and Wait (2009) have highlighted the importance of social ties in bereaved spouses’ ability to cope with their loss. Paradoxically, for participants in the current study, the provision of social support decreased substantially after the spouse’s burial. This holds implications for bereaved spouses’ ability to cope with their loss in the intermediate term, once support is not as forthcoming as it was in the earliest stages of bereavement. Indeed, participants reported being overwhelmed by grief in the days following the funeral service, once everyone who had come to pay their respects and to support the bereaved had left.

It is, however, worth noting that in the broader context, four of the participants highlighted their religious communities and figures as an important coping resource for a sustained period after spousal death. Kokou-Kpolou et al. (2017b, p. 769) have conceptualized such social support received within religious contexts as “religious community support,” thereby distinguishing it from types of social support provided in other contexts. The study findings suggest that, relative to other sources of support beyond their immediate family contexts, religious communities are in a unique position to provide varied, accessible, and consistent support to bereaved congregants at various stages of their bereavement.

The only male participant in this study reported that his daughter was his only source of emotional support after the burial. As with this study participant, Daggett (2002) found that the support that spousally bereaved men received was generally short-lived. Implications of the current study finding are especially concerning, considering Kokou-Kpolou et al.’s (2020) finding that bereaved male Togolese participants were at a higher risk of experiencing prolonged grief disorder, compared to spousally bereaved women. However, the current study participant’s receipt of emotional support from his daughter only might have coincided with the fact that she was the only one residing with the participant at the time, thereby automatically assuming the dual role of providing structural and emotional support. Another participant, whose niece resided with her for the primary purpose of companionship after her husband’s death, also reported that her niece was a valuable source of support for her, also soliciting emotional support from her (the bereaved spouse’s) friends when necessary. Therefore, it would seem that the bulk of the responsibility to provide emotional support automatically fell on those in close physical proximity to the bereaved spouses, and that the bereaved drew from the most accessible support structure, who in most cases, were their children. Beyond this, participants received emotional and some instrumental support from religious figures, friends, siblings, and other family members who were not residing with them.

### Implications of Spousal Death on the Functioning of Bereaved Spouses and Families

The need particularly for emotional support was demonstrated by participants’ recommendations of counseling and peer support to help not only the bereaved spouses, but also other family members to come to terms with their loss. Specifically, participants experienced or anticipated a change in family dynamics, characterized by a changed, strained relationship between the surviving spouse and their children. In addition to the loss experienced by each family member, spousal bereavement leads to a major change in the family structure. The death of a spouse or parent necessitates, among others, adaptation by all family members to the surviving parent’s sole parental authority and parenting style in this capacity. A change in family dynamics, with parental authority and style as key variables, has been documented for single-parent families after divorce (Lazar et al., 2009). A participant in the current study stated that children experience their own respective losses, which typically go unacknowledged by those rallying around the surviving spouse. The negative, short- and long-term effects of a parent’s death on children’s functioning and psychological health have been widely documented (e.g., Berg et al., 2016; Meyer-Lee et al., 2020). Grief relating to parental death, coupled with the relationship with the surviving parent, and the surviving parent’s own intrapersonal state and grief course, may have a complex set of implications for family dynamics following a spouse’s death. These intersections highlight the need for counseling or psychosocial support for individuals other than the bereaved spouse within the family context.

With the loss of a spouse, participants experienced loss of various forms of structural and functional support that are inherent to the marital social structure (Uchino, 2004). This affected various aspects of their functioning and their ability to cope with their new circumstances, in addition to spousal death. Several events and contexts foregrounded spousal loss and compounded their grief. These included running the household, dealing with life difficulties, and financial struggles. Regarding participants’ self-reported financial struggles, studies have shown links between reduced post-bereavement income and psychological distress (Cacciatore et al., 2016), and between financial instability after a spouse’s death and life satisfaction (Bratt et al., 2016). In the current study, these instances necessitated increased self-reliance and participants’ assumption of multiple roles within their households. These findings are accounted for in Stroebe et al.’s (2005) dual-process model, in its reference to bereaved spouses’ concurrent contention with loss- and restoration-oriented stressors, characterized by dealing with the loss of their spouses, while assuming new roles and identities, and facing novel circumstances. In addition to missing companionship, one participant reported having unmet intimacy needs particularly in the earlier stages of her widowhood. The latter was a noteworthy acknowledgement, considering the stigmatization of widows’ sexuality. As elucidated by Nyanzi (2011, p. 378), “[w]idowhood is socially constructed as an asexual period” in patriarchal societies. This societal standard might also explain this acknowledgement by only one of the participants.

### Withdrawal of Support, Social Isolation, and Stigmatization During Bereavement

Some in-laws withheld support for the bereaved spouse, accompanying this with varying degrees of hostility. A participant’s in-laws believed that she had played a role in her husband’s death. Widows are often assumed to have killed their husbands, whereas a similar belief is not routinely held about widowers (Manyedi et al., 2003; Ndlovu, 2015, unpublished). The assumption might be that, because she presumably does not have assets of her own, a woman would have a vested interest in, and benefit materially from her husband’s death. Indeed, Dlukulu (2010, unpublished, p. 15) has reported that 80% of widows who visited her clinical practice reported that “their in-laws blamed them for the deaths of their husbands with the objective of gaining financially from their deaths.” Despite women’s economic activity, contributions to their households – both economic and non-economic – and growing economic independence especially in urban contexts in South Africa, the view that household economies are the sole preserve of men and that women’s socio-economic positions within households are by default subordinate to those of men remains (Murugani et al., 2014; Ndlovu, 2015, unpublished; Owusu, 2018, unpublished). In this study, this was demonstrated by one participant’s in-laws withholding their emotional and financial support when her husband died, on the grounds that she alone had benefited financially from her husband while he was alive. This position disregarded the fact that the participant was also employed while married and also contributed financially to the household. While not overtly demonstrated through practices such as widow disinheritance and eviction (Young, 2006; Murugani et al., 2014; Ndlovu, 2015, unpublished; Owusu, 2018, unpublished), there are persisting assumptions of women’s inherent lack of economic power in the context of marriage, accompanied by their disempowerment and derision after spousal death within family and broader contexts.

In the context of less proximal relationships, while others provided material and moral support when needed, another participant found her employer to be disturbingly insensitive regarding her loss. South African labor law makes provision for at least 4days of family responsibility leave per year for employees, which can be taken up in the case of bereavement (Ainslie, 2018). This, however, does not take into consideration the fact that grief might affect an employee’s ability to function optimally in the workplace. This necessitates more compassionate treatment by employers, after spousal death. Naidoo (2010, unpublished) argues that employers have an opportunity to provide much-needed psychosocial support to bereaved individuals by establishing bereavement support programs in the workplace. This is especially considering that individuals spend a substantial amount of time in the workplace, and that grief can affect not only bereaved individuals’ own work performance, but also their relationships with colleagues and their affect. From the African perspective, allotment of a limited period for grieving, so the bereaved can promptly return to work following bereavement, is impractical, as it prevents individuals from performing important “rituals that eternally connect them to the deceased” (Baloyi and Makobe-Rabothata, 2014, p. 236).

Participants also reported that their navigation outside the home was largely limited to going to work and performing other essential activities, due to mourning traditions. Arguably, this minimizes the support that bereaved spouses can access and might predispose them to poor psychological health, compound their sense of loss resulting from the spouse’s death, and exacerbate their grief. Previous studies have shown that bereaved spouses are indeed susceptible to poor psychological wellbeing (e.g., Sekgobela et al., 2020). The study participants’ recommendations of counseling and peer support for bereaved spouses might have stemmed from their awareness of this susceptibility. A participant requested concession to attend church services halfway through her 6-month-long traditional mourning period. In relation to this, at least half of the study participants reported that fellow congregants and attending church had helped them come to terms with their loss. Other than support provision, Stroebe et al. (2005) posit that distraction from the loss is an important coping mechanism, as exclusive focus on the loss would be psychologically exhausting. Therefore, bereaved persons engage in adaptive grieving by oscillating between confronting and avoiding the loss through either focusing on the loss or “get[ting] on with other things” (Stroebe et al., 2005, p. 52) at different points of the grieving period. The role of distraction as a coping mechanism by bereaved spouses within the dual-process model has been acknowledged by Kokou-Kpolou et al. (2020) in a study on a Togolese and French sample. Along with the current participants’ reports, these observations illustrate the importance of the support or distraction from the loss that bereaved spouses can access outside the home, which they are systematically barred from seeking out during the traditional mourning period in the study context.

Participants reported being stigmatized during their mourning periods. Stigmatization seemed tied to bereaved spouses being dressed in traditional mourning attire (*thapo*) and the view that they carry bad luck and can contaminate others with the shadow of death (*sefifi*) accompanying them, until after their cleansing. Contrary to reports of bereavement rituals positively contributing toward bereaved persons’ psychological wellbeing (Msimanga-Ramatebele, 2008, unpublished; Makgahlela et al., 2021), participants reported being negatively affected by the stigmatization associated with distinctive mourning clothes and widowhood, in general. This study’s only male participant had a shorter mourning period. Other participants, including the male participant, unanimously noted that widows were typically allocated a longer mourning period, sometimes out of spite. In contrast, widowers are typically exempted from long mourning periods and wear *thapo* or mourning attire that does not readily distinguish them as bereaved. The indigenous knowledge consultant’s observation in this regard is compatible with that of Ramphele (1996, p. 99), who noted that, through her mourning attire, “[the widow’s] body becomes a metaphor for suffering.” These observations point to differential, systematic treatment of bereaved persons on the basis of gender, with widows at a distinct disadvantage. The ostracization and generally poor treatment of widows in particular, have been widely reported in many African societies, including South Africa (Manyedi et al., 2003; Idialu, 2012; Mathonsi et al., 2016; Nyangweso, 2017).

### *Botho/Ubuntu*: Relationality in the Context of Spousal Bereavement

With regard to the dimensions of relationality, as pertaining to *botho/ubuntu*, participants’ experiences of social support during bereavement entailed cooperation, support, rapprochement, compassion, deference, and love on the one hand; and on the other, lack of support, stigmatization, and ostracization. The themes demonstrated both positive (e.g., co-operation, care, compassion, and love) and negative (e.g., lack of co-operation, discord, stigmatization, ostracization, and discrimination) treatment of bereaved spouses in various communal contexts, mainly as informed by communally constructed rites, values, and belief systems. The former set of experiences would ordinarily be attributable to the conventional conceptualization of *botho/ubuntu* (LenkaBula, 2008; Metz, 2011; Ramose, 2015). However, the communal construction and pervasiveness of the ill treatment of bereaved spouses, especially widows, suggest these practices to be as integral to *batho/abantu* (i.e., humans), collectively, as is the positive treatment. This perspective supports Mboti’s (2015) conceptualization of the relational aspect of *botho/ubuntu* as multi-faceted and not limited to harmonious relations, while disregarding disharmony. Broadly, this conceptualization of *botho/ubuntu*, as illustrated in the study findings, is compatible with findings in previous studies, showing that disharmony – especially the perception of covert disharmony – is inherent to interdependent socio-cultural contexts (Adams and Dzokoto, 2003; Liu, 2020, unpublished). The sense of disharmony in these contexts has been attributed to limited relational mobility or the perceptibly inescapable connection that individuals have with relational others in interdependent contexts (Liu, 2020, unpublished).

Kitayama et al. (2010) have found wellbeing to be associated with the avoidance of relational strain among the Japanese, whose cultural context promotes interdependence. These authors further posited that a mismatch between one’s psychological inclinations and prevailing cultural expectations or norms could be detrimental to health and wellbeing. In the current study, bereaved spouses’ adherence to culturally mandated practices, such as relative seclusion during the mourning period, and ostensibly passive contention with ill treatment by in-laws or others in the broader social context, might have been informed by their need to actively avoid relational strain or going against norms. However, these practices compromised the bereaved spouses’ psychological wellbeing, as discussed in the section on “Withdrawal of Support, Social Isolation, and Stigmatization During Bereavement.” This reveals a tension between bereaved spouses’ personal wellbeing as a result of having their personal bereavement needs met, and a sense of wellbeing stemming from their adherence to norms that, paradoxically, compromise their personal wellbeing.

This study broadens our understanding of interdependent socio-cultural contexts, by primarily demonstrating that, even in the context of bereavement, where resources are often maximized to provide the necessary support to those bereaved, social support is not indiscriminately provided to bereaved spouses. The study has revealed varying contexts and sources of disharmony, and new insights have emerged regarding implications of such disharmony for the bereaved in a southern African interdependent context. The current findings add a different dimension to those in existing literature, regarding the nature of disharmony, alongside harmony, in interdependent socio-cultural contexts.

## Limitations of the Study

More indigenous knowledge experts could have been consulted, based on bereaved spouses’ self-identified ethnicities, so that the mourning practices of non-Batswana participants could be situated within cultural contexts that are relevant for their specific ethnicities. However, South African studies conducted in different regions on different ethnic groups have shown striking similarities between the groups’ mourning rites. Moreover, despite participants’ diverse ethnic backgrounds, mourning rites and practices were, in fact, similar across the study sample. This precludes the possibility that this study did not adequately represent non-Setswana conceptualizations of mourning rites.

The current study sample comprised individuals who had been spousally bereaved for 2–12years. Grief is a dynamic phenomenon and the participants’ experiences or recollection thereof following a spouse’s death likely varied, as a result. Further, the sample primarily consisted of elderly and female participants, which prevented informed juxtaposition of participants’ experiences by gender or age.

## Conclusion

Spousal bereavement was shown to have short- and long-term implications on various aspects of surviving spouses’ lives and wellbeing, including personal, socio-economic, familial, and social. The role of social support as one of the bereaved spouses’ core coping resources illustrated the importance of social networks during bereavement. However, the social support that was provided to them by non-members of their households soon after bereavement gradually diminished, later becoming largely accessible through their religious communities. Moreover, during and after their mourning periods, bereaved spouses experienced poor treatment, negative attitudes, stigmatization, and ostracization in their family and broader social contexts. In relation to *botho/ubuntu*, the bereaved spouses’ relational experiences were, from a value perspective, both positive and negative, entailing both harmony and conflict, co-operation and lack thereof, and compassion and love, as well as indifference, stigmatization, and ostracization. These findings are compatible with existing literature on the existence of disharmony in interdependent socio-cultural contexts.

The study findings highlight a need for various stakeholders, including religious and civic organizations, and public health facilities such as community clinics, to devise ways to better support bereaved individuals and their families. Measures could include standard provision of pastoral counseling to bereaved families in religious organizations, the provision of grief counseling to bereaved spouses and families in community clinics, and the establishment of peer support groups within these contexts and in broader communities, to maximize avenues of social support for bereaved spouses. Civic organizations could also play a key role in helping change the harmful attitudes toward bereaved spouses and sensitizing the wider public toward their cause. Given the ever-looming threat of disinheritance, dispossession, and victimization particularly for widows following their spouses’ deaths, there is a need for the strengthening of legislation and for effective policy implementation to protect their rights in the family and broader contexts.

## Data Availability Statement

The raw data supporting the conclusions of this article will be made available by the author, without undue reservation.

## Ethics Statement

The studies involving human participants were reviewed and approved by the Faculty of Humanities’ Research Ethics Committee, University of Pretoria. The patients/participants provided their written informed consent to participate in this study.

## Author Contributions

TT: study conception and design, data collection, analysis, and interpretation of results, and manuscript preparation.

## Funding

Publication of this paper was partially supported by the University of Pretoria’s Open Access Fund.

## Conflict of Interest

The author declares that the research was conducted in the absence of any commercial or financial relationships that could be construed as a potential conflict of interest.

## Publisher’s Note

All claims expressed in this article are solely those of the authors and do not necessarily represent those of their affiliated organizations, or those of the publisher, the editors and the reviewers. Any product that may be evaluated in this article, or claim that may be made by its manufacturer, is not guaranteed or endorsed by the publisher.
